# Indents

**Published:** 1913-01-15

**Authors:** 


### INDENTS.

**☞Brief Articles of Technical or Scientific Value will receive notice and comment. Contributions invited.**

*Effects of Treatment.* Of course the cause in these cases like the preceding systemic causes is beyond the reach of the dentist, for, like the physician, he cannot turn back the hands on the clock of time and, therefore, the benefit derived from his treatment will be an improvement in the local conditions more or less permanent according to subsequent care. Moreover the amount of improvement will be just in proportion to the extent to which local conditions are responsible; if local conditions are largely responsible and impaired vitality very little, the improvement may be so considerable as to be called a "cure." If the local conditions are very little responsible and impaired vitality very much the improvement will be very little and short lived.

I am aware that at present impaired vitality is not recognized as a cause of pyorrhea, but dentistry is the only field of pathology that I know of that does not recognize diminished vitality as a factor in disease.—*Items of Interest.*

*Cut Teeth from Throat.* Snatched from what seemed certain death and restored to the health which she enjoyed until a year ago by a remarkable surgical operation, Miss Mary Cremins, 18, left Suydenham hospital, where she was successfully operated on several days ago.

More than a year ago, unkown to herself, the girl swallowed several closely bridged false teeth, which lodged in the lower part of her throat. Surgeons had insisted that her trouble either was cancer or a tumor. Some of them had told her an operation was hopeless.

Unable to eat and wasting away, Miss Cremins went to Suydenham hospital a week ago. The house physician had several X-ray photographs taken. The teeth in her throat were apparent in the photograph and it was decided to operate.

*Dentistry Producing Disease of the Gums.* In 1886 I published a paper, (I think the *Cosmos*), making the statement that dentistry was producing more disease of the gums than any other one thing, and I have worked on that line for forty years. The principle of saving teeth is wrong. Every time we put the rubber dam or a clamp on a tooth, or wedge the teeth apart for the purpose of filling, or injure the gum margin with an instrument, we set up an irritation that eventually, whenever the system is out of order, that is to say, when the blood is not perfectly pure, will affect the alveolar process. If we cannot treat a tooth and save it without irritating the alveolar process and the gum margin, it is much better to remove that tooth; it is a quicker way of getting rid of it and the patient would have a healthier mouth, then he could possibly have under our present methods of operation.—E. S. Talbott in *Journal A. M. A.*

*The Turkish Bath.* I have been interested in the Turkish bath, that is in its use, theoretically and personally for forty years, from the fact that a brother-in-law, an old physician, Dr. Moses P. Hanson, of Milwaukee, conducted a Turhisk bath sanitarium for twenty-five years. I can give some information upon the subject which may interest the readers of the *Brief.*

1. *What is a Turkish bath?*—It is the application of dry heat to the naked skin with temperature ranging from 150 degrees to 220 degrees Fahrenheit.

2. *What is the theory of the effects of the application of high temperature to the human body?*—When the body is exposed to high temperatures nature demands relief, and it comes through evaporation of moisture from the skin. In tropical climates, where little or no clothing is worn, perspiration is excessive and sun-strokes are unknown.

3. *What is the modus-operandi of the bath*?—The patient, divested of clothing, lies down on a lounge with sheet wrapped around, and remains a half hour or more until thorough perspiration has resulted, drinking all the water he wishes. If he should possibly feel faint, he goes to the cooling room a few minutes and returns to the hot room. Some persons' skins do not respond at the first trial owing to inactive skin, and the bath is the remedy for this. From the hot room one goes to the shampooing room, lies upon a marble slab and the operator gives a thorough massage of the whole body, then scrubs with brush or hair mitten, then to the shower, first hot and gradually colder; some take the plunge. The patient is then wiped and goes to the cooling room lies upon a lounge wrapped in a sheet until cool and dry, then dresses.

After this process there is far less liability of taking cold than when he entered, for although there has been excessive perspiration, the cooling process has closed the pores, but the circulation has been restored to the surface and thus fortified against taking cold.

A reprehensible practice is sometimes resorted to or following the hot room with the steam bath, thinking there is an increased perspiration while in fact there is none, the body being immersed in vapor which condenses on the skin; the individual thinks it is perspiration, but he does not really perspire.

4. *What is the result of a Turkish bath?*—Three things absolutely essential to perfect health: First, at the demand for relief from the heat nature opens the pores, which perhaps have been long inactive, resulting in other organs taking on extra duty, and now the poisonous materials are being eliminated through nature's sewerage system, the pores. Second, in order to provide the perspiration the blood is called to the surface, thus equalizing the circulation from head to feet. Third, in doing this all congested conditions are relieved to an extent greater than by any other means.

5. *What is the difference in effect upon the human system as to the use of dry or moist heat?*—The steam bath or hot-water bath while accomplishing some good, does not cause perspiration until after leaving, as the body is already submerged in moisture and is weakening, while the hot air bath is a real tonic. Dr. Hanson in his long experience with the bath found that the very weak patient recuperated more rapidly under two baths a day than under one, showing the hot air bath is not weakening. The box cabinet for home use is a good thing, although not as serviceable as the regular system. If one is taking the baths as a remedial agent, taken daily are far more serviceable than at intervals. Some will say, "Why, you don't mean to say the bath will cure all diseases'." Why, it simply places the entire system in a condition to relieve itself of diseases to an extent nothing else does.

For the dentist who has stood at the chair all day, there is nothing will refresh him as a thorough Turkish bath. This can be taken immediately after eating, as it aids rather than retards digestion as a hot water bath is supposed to do.—Dr. Haskell in *Brief*.

*Gagging During Impression Taking.* By wiping spirits of camphor on the roof of the mouth and on the tongue, and directing the patient to swallow, you can take an impression of the mouth without the patient gagging and with perfect comfort.—Dr. Watkins, *The Journal*, June, 1912.

*His Wisdom Teeth at Fault.* De Forrest McLin, son of Dr. G. H. McLin, and now a student at Winona Academy, Huntington, Ind., is thankful that he has no more wisdom teeth coming. Some weeks ago he became suddenly deaf. He attributed it to an accident while bathing. He came home and consulted specialists in an attempt to recover his hearing. The trouble was coincident with the appearance of a wisdom tooth, which caused much inflammation, and he was taken to a dentist, the tooth extracted and he could hear as well as any one. A few days ago he seemed to be going blind in one eye and he thought of the other wisdom tooth. A dentist was consulted, the pliers were applied and his defect of vision was corrected without the use of glasses.—*Dental Brief.*

*Smoking in Winter Injurious.* According to a London physician, smoking has a worse effect on most people in winter than in summer, and he advises all smokers who find their health and mental faculties impaired in winter for no apparent reason to accept tobacco as the explanation and to cut down their smoking during colder months.

Tobacco, he said, is a very powerful drug, and cannot be consumed in large quantities without producing a certain effect on the heart. It also has opposite effects on the heart. First it slows the action, then it quickens it; and it is this constant reaction which eventually produces smoker's heart.

It must be remembered that during the winter the heart has a great deal more work to do than in summer, for the cold causes the blood vessels to become small and pinched. It is thus far less able to bear the extra strain put upon it by smoking, and the reaction produced by tobacco is felt to a much greater degree. And you cannot continue overworking your heart without unpleasant results.

Since the effect of tobacco varies with the individual, concluded the physician, it is not invariably the case that smoking has a worse effect in winter than in summer. But it undoubtedly is so with a large proportion of people.—*New York Sun.*

*Vitiated Saliva.* The state of the mind of a patient has much to do with changing the salivary secretions and in destroying the teeth. In my own mouth for thirty years I had no cavities in my teeth to fill, and there was no decay going on. I was absolutely immune from decay of the teeth. My son became sick and died, great sorrow came into my family, and my teeth ever since have been decaying like wildfire. Every tooth in my mouth is getting very sensitive at the necks, and decay is going on, so that it is almost discouraging to try to have the cavities filled. I have seen the same thing in business men who have got into financial or other difficulties, where their teeth have been absolutely free from decay, but as soon as financial trouble came on their teeth began to decay rapidly. I had never heard this phase of the subject discussed before, and I only thought of it when it came home to myself. I believe it is a prominent cause of a change in the secretions of the mouth which is destructive to the teeth.—Dr. J. N. Crouse in *Review*.

*Acid Saliva.* As you probably know, some of you, I have been doing some work for years along the lines of acid saliva, and I have been working along that line in a quiet way ever since I read a paper before the Chicago Odontological Society two or three years ago. About six years ago I went through a process of financial disturbance, and up to that time I was like Dr. Crouse—had no trouble with my teeth, but after a siege of carbuncles my teeth went to pieces. In my practice I have observed a number of similar instances in persons who were under high nervous tension; one a woman occupying an important position, and Board of Trade men, railroad despatchers, and men of that class. I examined their buccal secretions, from the parotid glands, and found they were acid. I followed it up with a diet. The way I first discovered this was by washing the mucous membrane around Steno's duct with alcohol, inserting a tube and collecting the secretion from the parotid gland, and found it was as much acid, or more so, than the other secretions of the mouth or the entire saliva. There was a time when we supposed that the acid condition of the saliva was due to bacterial fermentation, but upon investigation we found that the secretion from the parotid gland was as acid as the mixed saliva in the mouth. To verify these statements we took certain amounts of saliva and tested them for the amount of acidity, tirtrated them, and took the same amounts of saliva and allowed them to stand in test tubes from ten or fifteen minutes up to several days, and found that the acidity was not increased. After reviewing the literature of everything I could find on the subject, my attention was called to this line of work by Professor Herter of the New York University, who requested me to conduct a series of experiments along this line and follow them out, and I have been following up the subject for three years. Every Monday night, if I am at home, I keep a record of the amount of urine I pass, its acidity, its specific gravity, and so on. I also take ten c.c. of saliva and tirtrate that for acid against phenolphthalein. I have dieted for a month on vegetables; I have dieted for a month on red meat and mixed diets, and I find I can control the urine absolutely by diet, but cannot control the saliva. If I go out to a banquet, and drink a little too much wine, it affects my saliva. Whiskey does not have any effect, but wine does. If I am under a high nervous tension or strain, the acidity of my saliva is increased. Some of these days, if I can find a little time and have the inclination to figure out these proportions, these tirtrations mathematically, I will have this work published for the benefit of the profession.—Dr. J. E. Hinkins in *Review*.

*Wax Inlay Pattern.* There are two methods of procedure after the cavity is ready. One by making a wax pattern directly in the cavity and the other by taking an impression of the cavity in modeling compound and making a die of amalgam in which the wax pattern for the inlay is made. There are advantages in both methods and sometimes one can be used more successfully than the other. The wax should be of sufficient hardness to break rather than to bend when chilled and yet soft enough to be moldable when warmed. Waxes of different degrees of hardness can be used to advantage in certain cases. In a deep proximo-occlusal cavity in a bicuspid or molar it is difficult to get the wax to place at the first pressure. This being a hard wax a softer wax can be added to the part by drying the surface and flowing it on where needed. The wax of harder consistency will act as a plunger to press the other to place and aid greatly in difficult cases.—E. D. Coolidge in *Review*.

